# Evaluation of thermal pattern distributions in racehorse saddles using infrared thermography

**DOI:** 10.1371/journal.pone.0221622

**Published:** 2019-08-26

**Authors:** Maria Soroko, Daniel Zaborski, Krzysztof Dudek, Kelly Yarnell, Wanda Górniak, Ricardo Vardasca

**Affiliations:** 1 Department of Horse Breeding and Equestrian Studies, Institute of Animal Breeding, Wroclaw University of Environmental and Life Sciences, Wroclaw, Poland; 2 Department of Ruminants Science, West Pomeranian University of Technology, Szczecin, Poland; 3 Faculty of Mechanical Engineering, Wroclaw University of Technology, Wroclaw, Poland; 4 School of Animal, Rural and Environmental Sciences, Nottingham Trent University, Southwell, Nottinghamshire, United Kingdom; 5 Department of Environmental Hygiene and Animal Welfare, Wroclaw University of Environmental and Life Sciences, Wroclaw, Poland; 6 INEGI-LAETA, Faculdade de Engenharia, Universidade do Porto, Porto, Portugal; Universidade Federal de Mato Grosso do Sul, BRAZIL

## Abstract

The impact of a rider’s and saddle’s mass on saddle thermal pattern distribution was evaluated using infrared thermography (IRT). Eighteen racehorses were ridden by four riders with their own saddle. Images of the saddle panels were captured at each of six thermographic examinations. On each image, six regions of interest (ROIs) were marked on the saddle panels. The mean temperature for each ROI was extracted. To evaluate the influence of load on saddle fit, 4 indicators were used: ΔT_max_ (difference between the mean temperature of the warmest and coolest ROI); standard deviation of the mean temperature of the six ROIs; right/left; bridging/rocking and front/back thermal pattern indicator. Incorrect saddle fit was found in 25 measurements (23.1%) with ΔT_max_ greater than 2°C. The relationships between rider and saddle fit as well as saddle fit and horse were significant (p<0.001). An average ΔT_max_ in rider A was significantly higher than in other riders (p<0.001). The right/left thermal pattern differed significantly from the optimal value for riders A and B; while the bridging/rocking thermal pattern differed significantly from this value for riders A, C and D (p<0.05). Front saddle thermal pattern was most frequent for rider A (41.5%), whereas back saddle thermal pattern was most frequent for rider C (85.7%). Measurement of the mean temperature in 6 ROIs on saddle panels after training was helpful in assessing the influence of rider and saddle mass on saddle fit. IRT offered a non-invasive, rapid and simple method for assessing load on thermal pattern distribution in race saddles.

## Introduction

A correctly fitted saddle must accommodate the changing shape of a horse’s back during variations in horse gait, from trot to canter and gallop, in addition to allowing the rider to remain balanced [1]. Traditionally, horses are ridden with wooden tree saddles which have panels filled with wool-stuffed flocking to allow distribution of the rider’s mass across the horse's back [2,3].

If a saddle is fitted incorrectly, this can result in detrimental consequences for the horse. This may include pain in the thoracolumbar region [2,4,5], tenderness and stiffness of the longissimus dorsi muscles [3], spine osseous pathology and muscle atrophy decreasing horse performance [6]. A saddle fitted incorrectly has been shown to cause an increase of imbalance in the horse’s motion pattern [7].

In a study presented by Meschan et al. [8], it was demonstrated that under poorly fitted saddles the load (rider and saddle mass) is distributed over a smaller area leading to pressure peaks compared to properly fitted saddles. The way a rider distributes weight on the saddle is an important aspect that determines whether a horse can move easily and freely under a rider.

Also, Fruehwirth et al. [9] indicated that the overall force applied by a saddle pad is approximately equivalent to the rider’s body mass while the horse is walking. However, as aspects of the horse’s gait change, the amount of force exerted by the rider also changes. During trotting, the force values increase to approximately twice the rider’s body mass, and at canter the force increases to 2.5 times that of the rider’s body mass.

Equine back-related problems can also be associated with a poorly skilled rider due to asymmetry of the rider’s position or lack of balance [3,10]. Peham et al. [11] showed that the rider’s training level can influence the interaction with the saddle and thus with the horse’s back. Rider and saddle mass influenced the overall extension of the equine back while riding.

A study presented by Cocq et al. [12] reported that the load (75kg) applied influenced the movement of the horses back while walking and trotting on a treadmill. Similar results have been presented in another study, where the rider influenced locomotion variables of a horse on the treadmill [13]. An experienced rider with a good seated position may improve a horse’s balance and stability [7,14], but the forces acting on the horse’s back may reach three times the rider’s mass and may thus represent an enormous stress [15].

Pullin et al. [16] and de Cocq et al. [17] utilised a variety of pressure mats that measure force applied on the horse’s back in order to provide a quantitative assessment of saddle-fit. Studies presented by Jeffcott et al. [18] and de Cocq [19] found a linear relationship between the rider’s body mass and the pressure under the saddle. The total pressure applied via the pad was closely correlated to the rider’s body weight [9,17].

Other studies have found infrared thermography (IRT) to be a valuable tool in non-invasive evaluation of saddle thermal pattern distribution [20–22]. Although thermal pattern is affected by various factors such as the horses’ gait [9], horse performance and training intensity [3,22], girth tension [23], saddle pads [6], overall impulsion and balance [24], age and body condition [20–22], or conformation [8,14,25], its relationship with load is unknown.

It is interesting to indicate effect of load on saddle thermal pattern distribution as IRT presents advantages in relation with pressure systems. In human literature, Yavuz et al. [26] indicated that contact pressure and friction in different foot regions can impact both pressure and skin thermal pattern. It has also been proven that there is a relationship between contact load and the increase of the foot temperature during walking. Higher temperature elevation correlated with higher contact load. Regions with less contact tended to show relatively lower temperature elevations [27].

Based on these findings, the current study aimed to investigate the influence of load on thermal pattern distributions in racehorses. The impact of load on saddle thermal pattern distribution was evaluated using IRT.

## Materials and methods

### Data collection

Eighteen Thoroughbred racehorses (6 mares and 12 stallions), all 3 years old, were used. The horses were clinically healthy, with no apparent back injuries or lameness. Clinical history was obtained from clinical case files to determine whether the horses had any prior history of back pain and/or had been treated by a veterinarian for back pain/spinal dysfunction. A standard physical examination of the musculoskeletal system of each horse was performed by an experienced equine clinician to confirm any clinical injuries. The examination evaluated movement to identify the type and degree of lameness and to perform flexion tests [28]. The examination of the thoracolumbar region included palpation and mobility tests [29].

All horses had a similar level of fitness and were trained daily for flat racing in a clockwise direction at Partynice Race Course (Poland) during the 2017 season. The horses were housed in individual stalls with common management and training regimes.

During daily training, horses trotted a distance of 1 km and cantered on the right lead at distances of up to 3000 m on a racing court (2150 m in length). The level of fitness was assessed using the speed achieved during canter. The horses were ridden by their usual riders (n = 4) and the study relies on every horse having similar training in distance and time length.

Four treed race uniformly flocked saddles, (saddle: I, II, III, IV), with mass (including stirrups and girth) between 3.6–4.5 kg were utilised during the study. Four experienced female riders (rider: A, B, C, D) with 3–4 years’ riding experience, mean body mass of 54.8 (±2.9) kg, Min = 51.0 kg, Max = 58.0 kg, and height of 158 (±2.9) cm participated in the study (Table 1).

**Table 1:** Saddle mass and rider body mass used in the study.

Saddle	Saddle mass (kg)	Rider	Rider body mass (kg)	Load: rider plus saddle mass (kg)	Number of training sessions
I	3.6	A	58	61.6	24
II	4.4	B	57	61.4	37
III	4.5	C	53	57.4	25
IV	3.7	D	51	54.7	22

Thermographic assessment of the saddle was conducted for each horse six times (three times per week at intervals of 3 days) over 2 weeks. Horses were tacked up in a standardised way using a single thin numnah made of polyester. The rider mounted inside the horse’s box with trainer help. To avoid any confounding effects of differences in tightening, saddles were always tightened by the same person (trainer) after the rider had mounted.

Horses underwent training which consisted of a 5-minute warm-up (walk), 5 minutes of trotting, and 20 minutes of cantering (2200 m) on the racing track. Horses were ridden in the same standardised way, which consisted of rising trot and two-point seat at canter. After training, horses were untacked in the stable and cooled down on an automatic horse walker for approximately twenty minutes.

On the examination day, each horse was ridden by a different rider. Rider A was using saddle I, rider B was using saddle II, rider C was using saddle III and rider D was using saddle IV (Table 1).

### Infrared thermography

Thermographic measurements were taken using an InfraTec® VarioCam HD Resolution infrared camera (uncooled microbolometer focal plane array, Focal Plane Array sensor size of 640 x 480, spectral range 7.5–14 μm, Noise Equivalent Temperature Difference of <20mK at 30°C, using the normal lens with IFOV of 0.57mrad, measurement uncertainty of ±1% of the overall temperature range, InfraTec Dresden, Germany).

**Fig 1:**
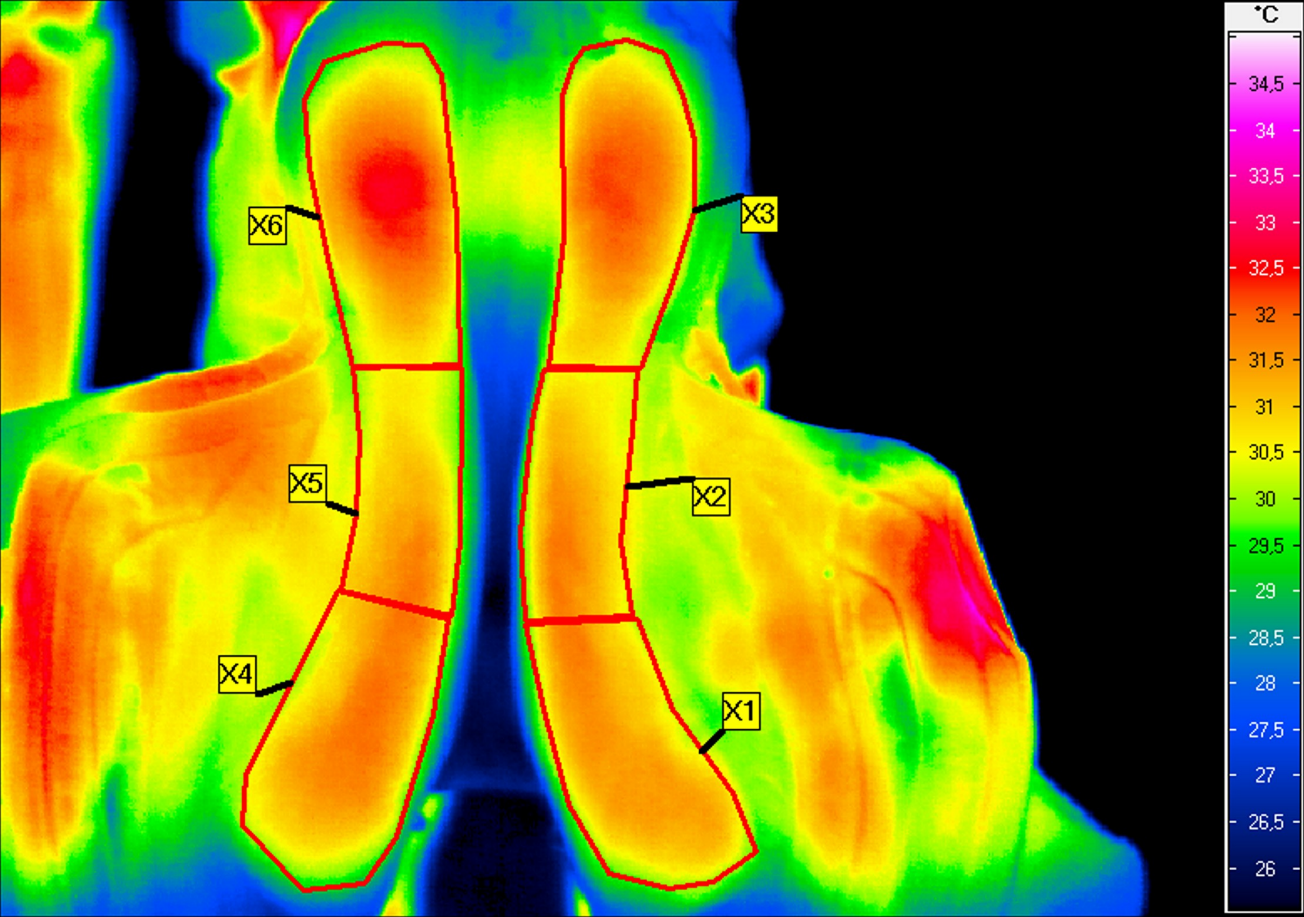
Thermographic image of saddle panels. Thermographic image of saddle panels taken immediately after untacking the horse, with the six regions of interest (ROIs) indicated: right front of the saddle (X1), right middle of the saddle (X2), right back of the saddle (X3), left front of the saddle (X4), left middle of the saddle (X5), left back of the saddle (X6).

The ambient temperature (Tamb) in the stable was measured with a TES 1314 thermometer (TES, Taipei, Taiwan). Thermographic images of the saddle panels were taken once, 2–3 seconds after untacking the horse (Fig 1) inside the box, while another person was holding the horse. The protocol for the thermographic examination was conducted as previously described by Arruda et al. [20] and Soroko et al. [22].

To minimize the effect of environmental factors, thermographic images of the saddle panels were always performed at the same place within an enclosed stable in the cordial. The distance from the saddle to the camera was fixed for all imaging at 1m, and the emissivity (ε) was set to 1 for all readings as per the protocol of Arruda et al. [20].

All thermographic imaging was performed by the same operator (MS). The ambient temperature was 20 ±3°C at the time that images were taken. This study was approved by the 2nd Local Ethical Committee of Experimental Procedures on Animals in Wroclaw, Poland (Protocol no. 44/2014).

### Data analysis

Six regions of interest were marked on the saddle panels as shown in Fig 1, and the mean temperature within each ROI was defined and calculated as follows:

X1 = the mean temperature in the region ROI1 (right side–front of the saddle).

X2 = the mean temperature in the region ROI2 (right side–middle of the saddle).

X3 = the mean temperature in the region ROI3 (right side–back of the saddle).

X4 = the mean temperature in the region ROI4 (left side–front of the saddle).

X5 = the mean temperature in the region ROI5 (left side–middle of the saddle).

X6 = the mean temperature in the region ROI6 (left side–back of the saddle).

The mean temperature was calculated using IRBIS® 3 Professionalo software (InfraTec, Dresden, Germany).

### Statistical analysis

A total of 108 thermograms associated with 31 horse-load combinations were analyzed (Table 2).

**Table 2:** Number of measurements (thermographic sessions).

Rider body mass plus saddle mass (kg)	Number of horse																	
	1	2	3	4	5	6	7	8	9	10	11	12	13	14	15	16	17	18
A+I = 61.6	0	2	0	5	0	1	0	4	5	0	0	2	0	2	1	0	0	2
B +II = 61.4	0	4	6	1	0	0	6	0	0	6	5	0	6	0	0	1	0	2
C +III = 57.4	0	0	0	0	0	5	0	0	0	0	0	4	0	0	5	5	4	2
D +IV = 54.7	6	0	0	0	6	0	0	2	1	0	1	0	0	4	0	0	2	0

**Fig 2:**
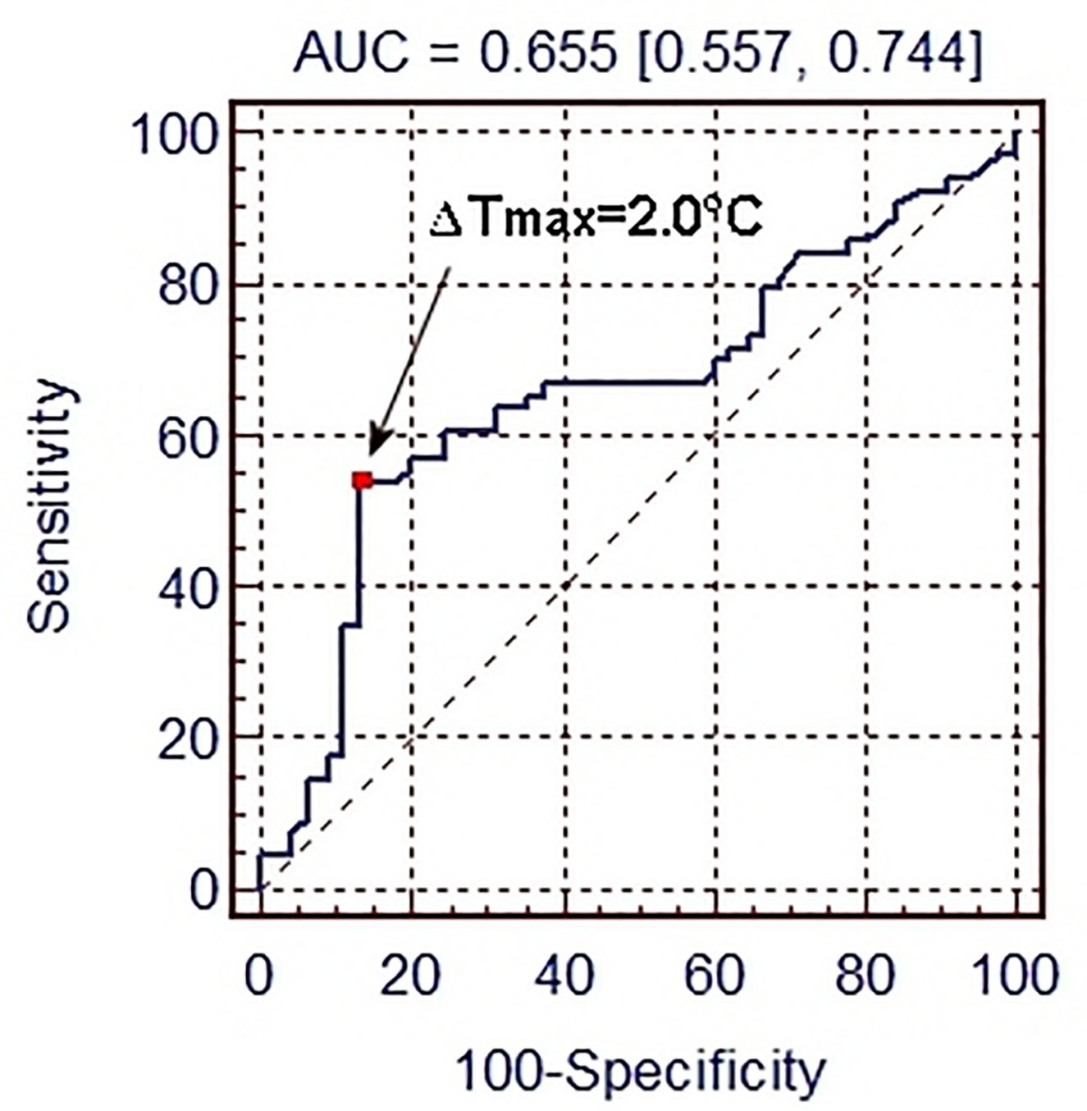
Receiver Operating Characteristic curve. Receiver Operating Characteristic curve for ΔTmax with indicated cut-off score ΔTmax = 2°C, for which sensitivity (SE) is 54.6%, specificity (SP) is 88.0% and area under curve is AUC = 0.655.

The following criteria were used to assess saddle fit:

- ΔT_max_—difference between the mean temperature of the warmest and coolest ROI. To identify threshold value Tmax> 2.0°C the Receiver Operating Characteristic (ROC) curve method was used (Fig 2), where correct saddle fit (symmetrical temperature distribution in six ROIs) met three criteria:
  -1°C<TI1<+1°C and -1°C<TI2<+1°C and TI3 = EL (Fig 2);
- SD_T_—standard deviation of average temperatures of six ROIs;
- TI1 –right/left panel thermal pattern indicator;
- TI2—bridging/rocking panel thermal pattern indicator;
- TI3—front/back panel thermal pattern indicator.

To evaluate thermal pattern distribution of the saddle, four indicators of temperature were taken into consideration (Table 3). To evaluate the effect of a rider on ΔT_max_, TI1, and TI2_,_ a one-way analysis of variance (ANOVA) was used (a normal distribution was verified using the Shapiro-Wilk W test and variance homogeneity using the Brown-Forsythe test; Tukey’s honest significant difference for unequal sample sizes served as a post-hoc test).

**Table 3:** Temperature indicators for saddle fit.

Temperature indicator	Temperature Index	Formula
ΔT_max_	Absolute saddle asymmetry index, i.e. the difference between the average temperature of the warmest (X_max_) and coolest (X_min_) ROI	X_max_—X_min_
TI1	Right hand thermal pattern—saddle places more pressure in the right panel.	(X1+X2+X3)-(X4+X5+X6) ≥ +0.5°C
TI1	Even thermal pattern—even thermal patterns between right and left panel	(X1+X2+X3)-(X4+X5 +X6) > -0.5°C and (X1+X2+X3)-(X4+X5 +X6) < +0.5°C
TI1	Left hand thermal pattern—saddle places more pressure in the left panel.	(X1+X2+X3)—(X4+X5+X6) ≤ -0.5°C
TI2	Bridging thermal pattern—both panels place pressure at the front and back.	(X2+X5)/2–(X1+X4+X3+X6)/4≤-0.5°C
TI2	Even thermal pattern—even thermal patterns between right and left panel	(X2+X5)/2–(X1+X4+X3+X6)/4<-0.5°C and (X2+X5)/2–(X1+X4+X3+X6)/4>+0.5°C
TI2	Rocking thermal pattern—both panels place pressure at the cantle	(X2+X5)/2–(X1+X4+X3+X6)/4≥+0.5°C
TI3	Front saddle thermal pattern—both panels place pressure at the front.	(X1+X4)≥(X2+X5)≥(X3+X6)
TI3	Even thermal pattern—even thermal patterns between right and left panel	(X1+X4)≥(X2+X5)<(X3+X6) or (X1+X4)<(X2+X5)≥(X3+X6)
TI3	Back saddle thermal pattern—both panels place pressure at the back.	(X1+X4)≤(X2+X5)≤(X3 + X6)

The significance of the differences in the TI1 and TI2 indicators from the optimal value (equal to 0) was tested with the Student’s t test. To analyze the relationship between the rider and saddle fit, the horse and the saddle fit as well as the rider and the TI3 category, a chi-square test of independence was applied. All the analyses were performed using the Statistica program (v. 12, StatSoft Inc., Tulsa, OK, USA). Statistical significance was declared with a 95% confidence interval.

## Results

**Fig 3:**
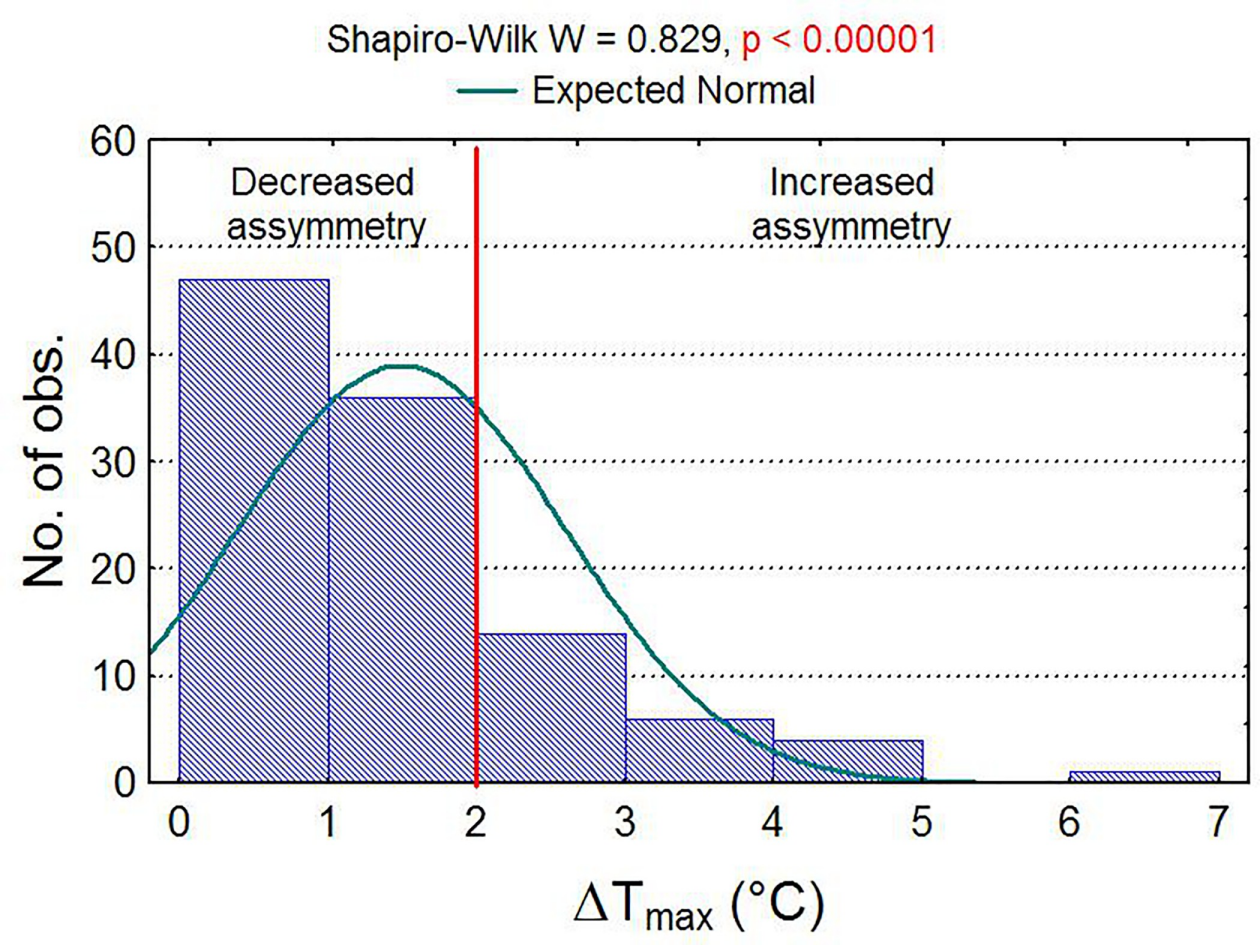
Histogram of maximum temperature differences. Histogram of maximum temperature differences ΔT_max_ at normal distribution and results of the normality test.

In 108 thermographic measurements of saddle panels, ΔT_max_ distribution was strongly asymmetric (Fig 3). Of the 108 measurements, there were 47 cases with a temperature range of 0°C to 1°C (43.5%) and 36 cases with a temperature range of 1°C to 2°C (33.3%). In 25 cases (23.1%) of interaction saddle–rider value of ΔT_max_ was above 2°C. Result of ΔT_max_ > 2°C indicates incorrect saddle fit.

Incorrect saddle fit, identified by asymmetric thermal pattern, was indicated in 11 cases (Table 4). Horses 4 and 9 (trained by rider A) each had 5 occurrences of incorrect saddle fit. When the horses were ridden by other riders, ΔT_max_ value was less than 2°C. Horses 2 and 14 had lower incidences of temperature asymmetry (3 cases each).

**Table 4:** Temperatures of incorrectly fitted saddle indicated with regions of interest (ROIs).

Set Horse-Rider	*n/N*	Mean (±SD) temperature in ROI (°C)						ΔT_max_
		ROI1	ROI2	ROI3	ROI4	ROI5	ROI6	
1-D	2/6	29.9 ± 5.2	29.6 ± 5.6	26.5 ± 6.6	30.0 ± 5.3	29.6 ± 5.7	26.8 ± 6.8	2.2 ± 1.2
2-A	2/2	33.1 ± 1.7	33.0 ± 1.8	31.1 ± 1.7	33.0 ± 1.8	32.8 ± 1.9	31.2 ± 1.7	2.0 ± 0.0
2-B	1/4	25.4	24.7	23.1	24.6	24.2	23.5	2.3
4-A	5/5	31.9 ± 0.7	31.1 ± 0.9	28.8 ± 1.6	31.8 ± 0.7	31.1 ± 1.0	29.3 ± 1.8	3.2 ± 1.0
6-B	1/1	33.4	33.0	31.0	33.5	33.1	30.9	2.6
8-A	3/4	32.8 ± 2.2	32.0 ± 2.5	29.4 ± 3.0	32.8 ± 2.1	32.6 ± 2.3	29.9 ± 3.2	3.5 ± 0.8
9-A	5/5	33.4 ± 2.1	32.9 ± 2.8	30.3 ± 3.5	33.4 ± 1.9	33.0 ± 2.5	30.7 ± 3.2	3.4 ± 1.7
12-A	2/2	33.8 ± 1.4	32.6 ± 2.0	30.1 ± 2.7	33.4 ± 1.6	32.7 ± 2.0	30.5 ± 2.6	3.7 ± 1.2
14-A	2/2	32.8 ± 2.1	32.0 ± 2.7	30.3 ± 3.3	33.1 ± 2.6	32.5 ± 2.9	30.9 ± 3.4	2.9 ± 0.8
14-D	1/4	31.2	30.5	28.2	30.9	30.9	28.8	3.0
18-A	1/2	27.8	26.4	24.3	27.8	26.4	24.5	3.5

N–total number of measurements, n–number of measurements with incorrectly fitted saddle (ΔT_max_ ≥ 2°C)

Horse 2 was ridden 3 times by two riders (A and B). Similarly, horse 14 was ridden 3 times by two riders (A and D). Rider A (61.6 kg–rider plus saddle mass) rode with an incorrectly fitted saddle on 20 separate occasions out of 24 training sessions, rider B (61.4 kg rider plus saddle mass)–rode using an incorrectly fitted saddle on two occasions out of 37 training sessions, rider C (57.4 kg rider plus saddle mass)–did not ride in an incorrectly fitted saddle out of a total of 25 training sessions, and rider D (54.7 kg rider plus saddle mass)–rode in an incorrectly fitted saddle 3 times out of 22 training sessions.

**Fig 4:**
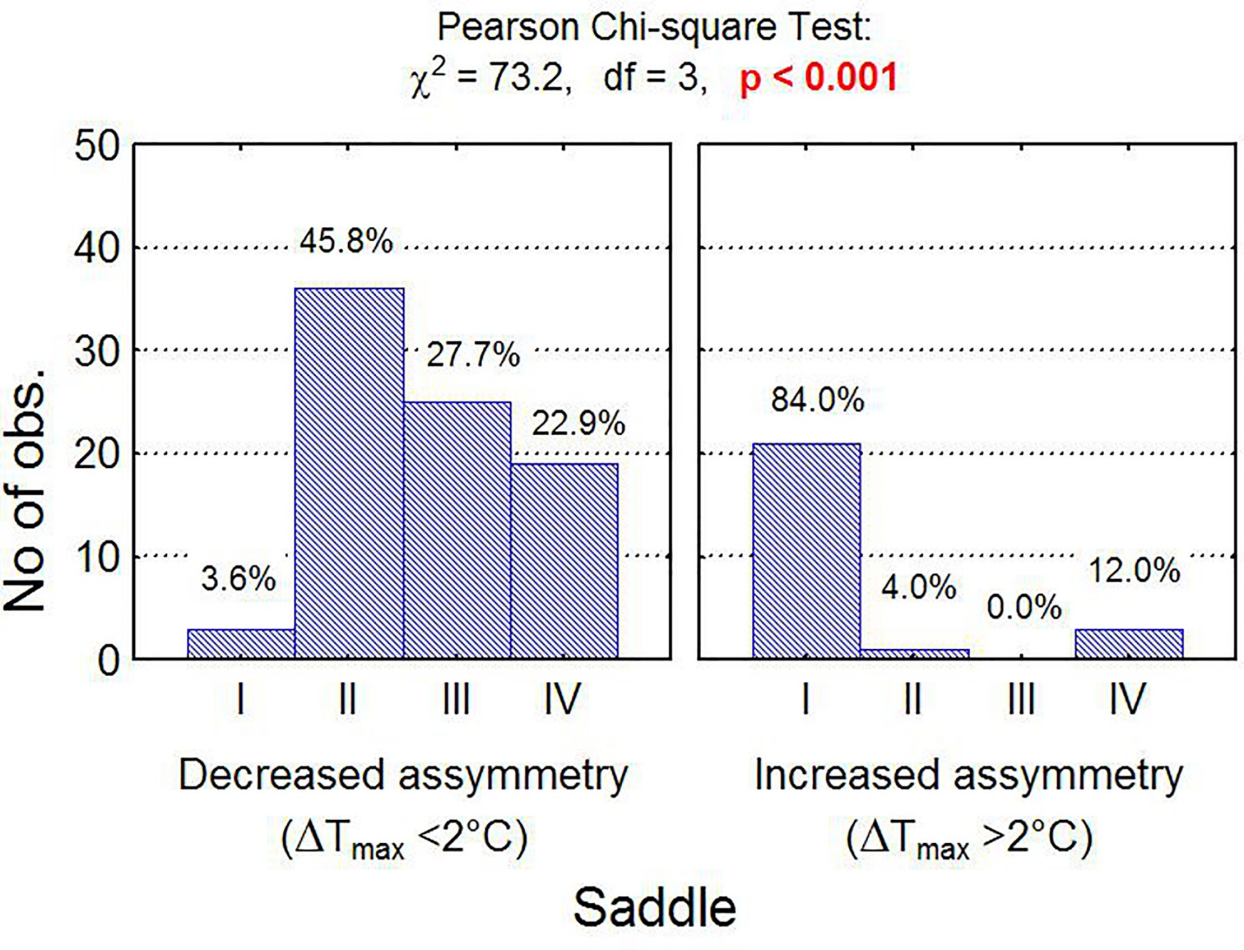
Number of training sessions in groups. Number of training sessions in groups differing with rider and saddle fit and independence test results.

**Fig 5:**
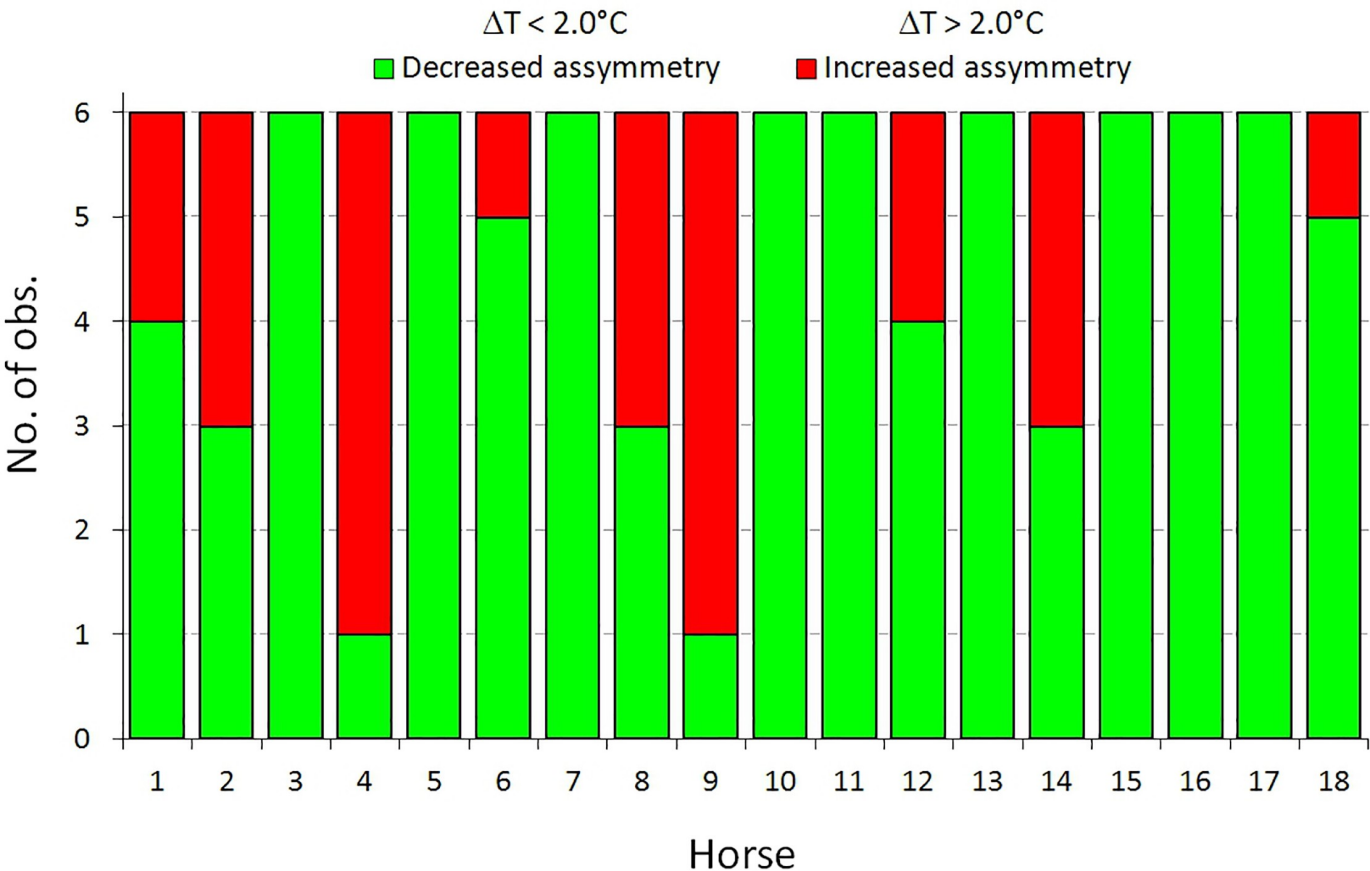
Number of training sessions in groups. Number of training sessions in groups differing with horse and saddle fit and independence test results.

Both the relationship between the rider and saddle fit (p<0.001; Fig 4) and the relationship between saddle fit and horse (p<0.001; Fig 5) indicated by ΔT_max_ were statistically significant. There was a statistically significant relationship between saddle thermal pattern distribution (saddle fit) and type of the horse. An unfitted saddle was more frequent for horses 4, 9, 2, 8, and 14 than horses 3, 5, 7, 10, 11, 13, 15, 16 and 17 (Fig 5).

**Fig 6:**
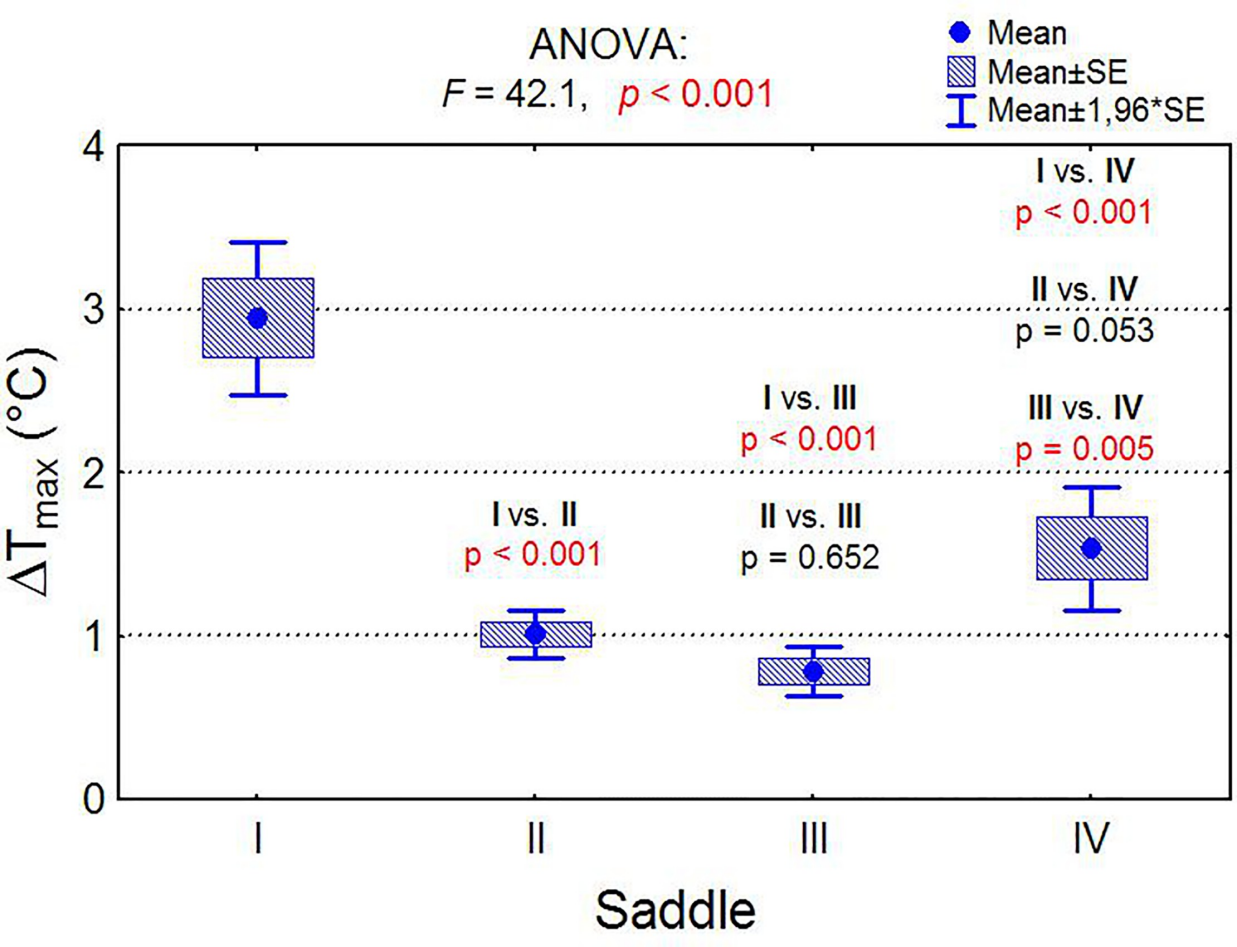
Comparison of the mean values of the temperature index. Comparison of the mean values of the temperature index ΔTMAX of four saddles and the analysis of variance results (ANOVA) and multiple comparisons (post-hoc tests).

As shown in Fig 6, the average ΔT_max_ value for rider A was significantly higher than that for other riders (p <0.001). A significant difference in ΔT_max_ was also observed between riders C and D (p<0.05).

**Fig 7:**
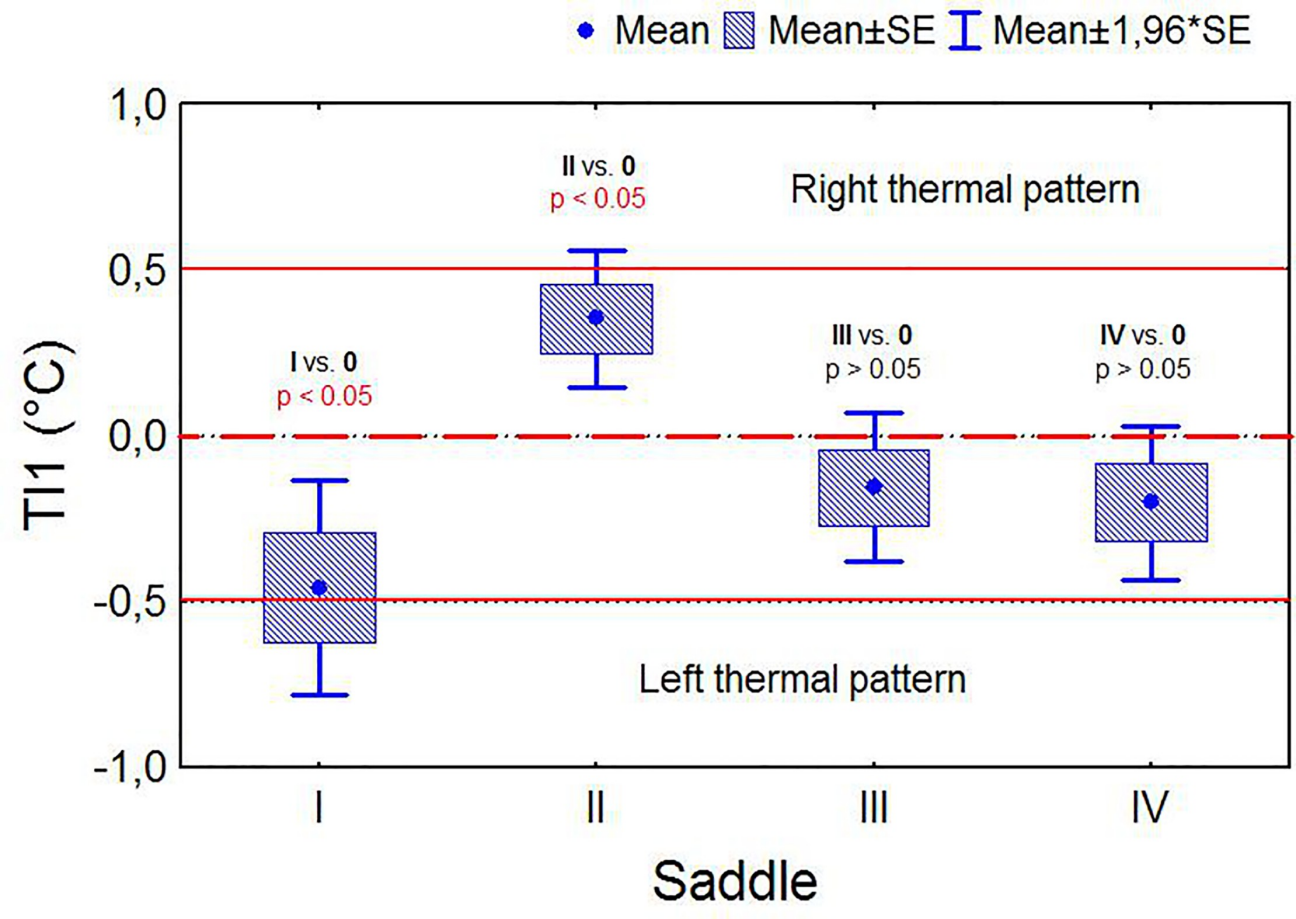
Comparison of the mean values of temperature indicators. Comparison of the mean values of temperature indicators TI1 (right/left panel thermal pattern indicator) for four saddles and results of significance tests (T-tests for single means).

**Fig 8:**
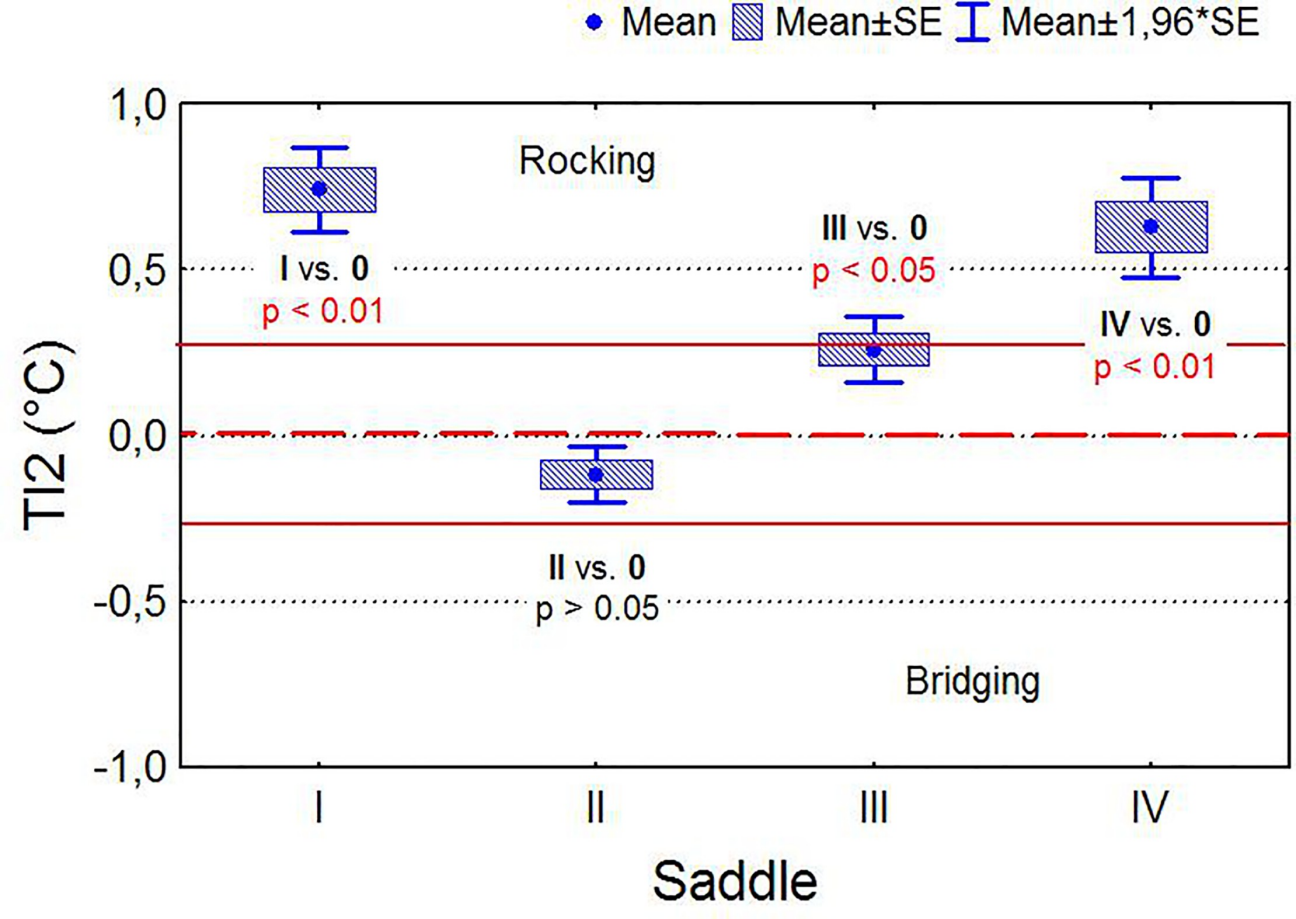
Comparison of mean values of temperature indicators TI2. (bridging/rocking panel thermal pattern indicator) for four saddles and results of significance tests (T-tests for single means).

Rider A exerted more thermal pattern distribution on the left saddle panel, whereas Rider B exerted more thermal pattern distribution on the right saddle panel; however, the other two riders consistently distributed their body mass symmetrically (Fig 7). Saddles I, III and IV ridden by riders A, C and D frequently presented a thermal pattern consistent with rocking, which occurred in the case of both a heavy (rider A) and light rider (rider D) (Fig 8).

**Fig 9:**
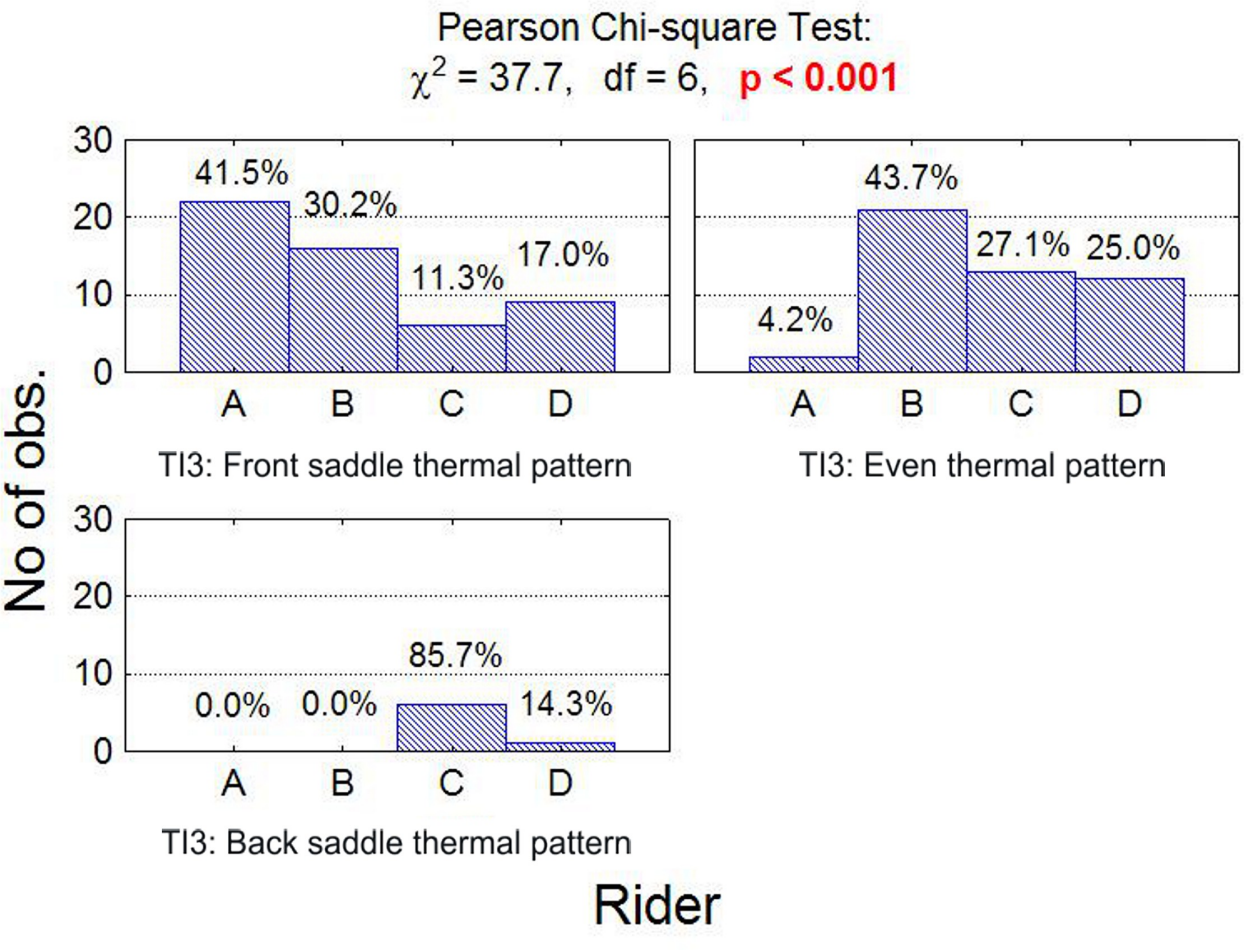
Number (fraction) of observations in subgroups. Number (fraction) of observations in subgroups that differ in the temperature index TI3 (front/back panel thermal pattern indicator) and saddle and the result of chi-square independence test.

A statistically significant relationship was found between front/back thermal pattern and the rider (Fig 9). A front saddle thermal pattern was most frequent for rider A (41.5%) and least frequent for rider C (11.3%), whereas back saddle thermal pattern was most frequent in rider C (87.5%) and least frequent in riders A and B (0.0%). Finally, an even thermal pattern was most often observed in riders B (43.7%) and C (27.1%).

## Discussion

IRT has been previously found to be useful for the assessment of saddle thermal pattern distribution in racehorses [20–22]. This study is the first to utilise ΔT_max_, which compares the difference between the mean temperature of the warmest and coolest ROI, for an assessment of saddle fit. The relationship between the rider and saddle fit indicated by ΔT_max_ was statistically significant, as was the relationship between saddle fit and horse.

A difference in temperature between the saddle panels above 2°C suggests incorrect saddle fit. Previous studies indicated temperature differences of 1°C in the diagnosis of pathological conditions [30–31].

In the current study, 25 (23.1%) of the 108 thermographic measurements had a temperature difference greater than 2°C. A study carried out on 51 jumping horses [32] reported that 35% of asymmetric heat distribution related to poor saddle fit was located in the thoracolumbar region. This is supported by a study presented by Arruda et at. [20] which reports that 55.8% of the jumping horses assessed using IRT had an asymmetric thermographic image of the thoracolumbar region after training.

Two horses from the study trained by one rider (rider A) had 5 occurrences of asymmetrical thermal pattern distribution. When the same horses were ridden by other riders, the ΔT_max_ value was less than 2°C. Possible stiffening and tensing from the rider could indicate difficulties with following the movement of the horse [33].

Physical or postural asymmetry of a rider results in asymmetric distribution of force via the saddle to the horse [34]. Persistent crookedness of a rider could potentially cause asymmetry in horse locomotion, resulting in an established asymmetric locomotor pattern and/or secondary pain, which can later lead to spine diseases in the horse [35–36].

It may be that the higher forces associated with a heavier rider and kit exacerbate the inherent asymmetry of the horse. It is possible that the saddle may not have fitted rider A, which could have caused the imbalance and resulted in uneven pressure distribution.

Saddles with ΔT_max_ values above 2°C presented with rocking/bridging, front/back and right/left thermal pattern. Bridging has been identified as a major problem in saddle fit, where loading on the horse’s back is concentrated in the front and back of the saddle [2] and it is potentially detrimental because it causes focal distribution of the rider’s weight, rather than distributing it evenly over a larger area [4].

However, in the current study only rocking pressure occurred with saddles I, III and IV ridden by riders A, C and D. A rocking thermal pattern occurred under both heavy (58 kg) and light riders (51–53 kg), which can suggest that the weight of the rider had no influence on saddle thermal pattern distribution. However, both Meschan et al. [8] and Belock et al. [37] indicated that pressure is more concentrated with poorly fitted saddles with heavier riders.

Rider A exerted more thermal pattern on the left saddle panel. This could be associated with the uneven weight distribution by the rider or by asymmetric musculature of the horse. Similar results were found in our previous study, with increased thermal pattern placed on the left side in loads between 45–50 kg and 51–55 kg [22].

Also, riders in English dressage saddles presented more left side load during riding in an indoor riding arena [9]. Another study indicated that left–right asymmetries were associated with the weight of the rider, the ratio of the horse’s weight and rider’s weight and the rider’s previous injuries.

Therefore, repetitive force applied by a heavy rider with an asymmetric position in the saddle is likely to place abnormal stress on the horse’s back on one side and contribute to the development of muscular asymmetries [3].

Visual assessment of “saddle slip” to one side could be a sign of hind limb lameness in ridden horses [14], asymmetry of the horse’s back or crooked riders [3]. Also, the riding skills and style of the rider may influence the symmetric movement of pelvis contributing to hind limb lameness during rising trot [38–39].

In front and back thermal pattern distribution, rider A most often presented front saddle thermal pattern (41.5%) and rider C, back saddle thermal pattern (85.7%). Putting more pressure on the front of the saddle can be the result of a two-point seat jumping position. The position of the rider is also influenced by the fit of the saddle relative to the horse [7,40] and the fit of the saddle relative to the rider.

A rider’s ability to ride in rhythm with the horse requires training and sensitivity to a horse’s motion. It is also influenced by the rider’s own symmetry, balance, fitness, pain, stability and correctness of position [10].

In the current study, IRT examination was able to identify uneven thermal pattern distribution linked to poor saddle fit. The observed asymmetry in surface temperature could have been caused by a number of factors, including movement patterns of the horse, asymmetry, rider balance and seat, and stiffness of both the horse’s and rider’s backs. Therefore, IRT examination is a useful diagnostic tool to conduct a preliminary assessment of potential problems with saddle fit.

The main limitation of this study is that IRT cannot distinguish among the effects of the rider, the saddle and the movements of the horse. To allow better discrimination in analysing thermal pattern distribution, future studies could use a pressure sensor matrix between the saddle and horse’s back and a sensorial solution for acquiring and recording the horse’s and rider’s activity. Therefore, further investigation is needed to assess the correlation between IRT examination and saddle pressure mats in saddle fit assessment.

## Conclusions

Measurement of the mean temperature in 6 ROIs on the saddle panels after training can be used in addition to other methods in assessing the influence of both rider and saddle mass on the thermal pattern distribution and therefore on the saddle fit.

Temperature indicators revealed that riding skills of the rider can have an influence on thermal pattern distribution. IRT offered a rapid assessment of saddle fit in addition to an objective method of assessing the rider’s seat. IRT is a simple method for assessing load on the thermal pattern distribution of race saddles in horses.
